# Hierarchical Channeled Graphitized Nanoarchitecture as a Diagnostic Platform for Maternal Fever Warning

**DOI:** 10.1002/advs.202521251

**Published:** 2026-02-23

**Authors:** Yiwen Lin, Ning Li, Heyuhan Zhang, Xufang Hu, Zhiqiang Liu, Chunhui Deng

**Affiliations:** ^1^ Department of Chemistry Fudan University Shanghai China; ^2^ Shanghai Key Lab of Reproduction and Development Shanghai Key Lab of Female Reproductive Endocrine Related Diseases Obstetrics & Gynecology Hospital of Fudan University Shanghai China; ^3^ School of Chemical Science and Technology National Demonstration Center for Experimental Chemistry and Chemical Engineering Education Yunnan University Kunming Yunnan China; ^4^ Center for Medical Research and Innovation Shanghai Pudong Hospital Fudan University Pudong Medical Center Department of Chemistry Institutes of Biomedical Sciences Fudan University Shanghai China

**Keywords:** graphitized carbon, hierarchical porous channels, intrapartum maternal fever, machine learning, nano‐diagnostic platform, N‐glycan extraction, precise diagnosis

## Abstract

The precision and effectiveness of nano‐diagnostic platforms rely on the deliberate design of advanced nanomaterials, aiming to address the clinical challenge, such as sensitive diagnosis of infectious chorioamnionitis‐associated fever (CAM) versus non‐infectious epidural‐related maternal fever (ERMF), while also expanding the scope of nano‐diagnostic technologies to include bio‐detection like glycomics, which are cohesively linked to disease but require complex procedures. Here, we designed a hierarchically channeled graphitized nanoarchitecture (HPGC‐Z67) as a novel nano‐diagnostic platform. HPGC‐Z67 features high graphitization and interconnected multi‐scale channeled structures that facilitate N‐glycan retention and mass transfer in expanded porous channels. Notably, compared to traditional protocol, this HPGC‐Z67 platform reduces processing time by about 25 min and cost by approximately CNY 30 per plasma sample, making it suitable for large‐scale clinical diagnostics. The HPGC‐Z67 nano‐diagnostic platform enables sensitive extraction of N‐glycan profiles from 150 plasma samples. Notably, two pivotal N‐glycans are identified, one sensitive to infectious fever (IF‐sensitive) and the other to non‐infectious fever (n‐IF‐sensitive), which together enable simultaneous differentiation of ERMF, CAM, and healthy controls with area under the curve values of 0.965 in the training set and 0.914 in the validation set, respectively. This HPGC‐Z67 nano‐diagnostic platform advances glycomics towards precise diagnostics and timely clinical intervention.

## Graphical abstract

This study presents a hierarchically channeled graphitized nanoarchitecture (HPGC‐Z67) as a novel nano‐diagnostic platform. It enables sensitive and efficient N‐glycan extraction from plasma, significantly reducing processing time and cost. The platform identifies pivotal N‐glycans to accurately differentiate between infectious and non‐infectious maternal fevers, demonstrating high diagnostic precision for clinical glycomics.

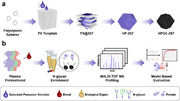

## 1 Introduction

Maternal fever during childbirth is a common symptom usually caused by two main factors. One potential cause is an adverse effect that occurs when local anesthetics are administered into the epidural space during epidural analgesia, which is a widely employed technique for labor pain management [1]. This epidural‐related maternal fever (ERMF) is a non‐infectious inflammatory process and affects approximately 20% to 30% of women receiving epidural anesthesia [2, 3, 4]. Another more serious form of fever is chorioamnionitis‐associated fever (CAM), an infection caused by bacterial invasion into the amniotic cavity [5, 6, 7]. CAM substantially raises the risk of neonatal early‐onset sepsis, meningitis, and long‐term neurological sequelae, alongside maternal risks such as endometritis and postpartum hemorrhage [8, 9, 10, 11, 12]. Given the similar initial clinical manifestations of ERMF and CAM, accurate and rapid diagnosis is essential for their timely and appropriate treatment [2]. However, current diagnoses mainly rely on serial maternal white blood cell counts and C‐reactive protein levels, which lack sufficient sensitivity and specificity. This often leads to delayed antibiotic therapy for CAM or unnecessary antibiotic exposure and invasive neonatal testing in cases of ERMF [7, 13, 14]. Therefore, there is an urgent need to develop novel diagnostic tools for accurate intrapartum fever risk prediction and reliable infectious origins distinction, thereby facilitating precise clinical interventions and improving perinatal outcomes.

Advanced bio‐detection nanomaterials are emerging as promising platforms for clinical diagnostic applications because of their easy fabrication and flexible adaptability [15, 16]. Recently, some potential nano‐diagnostic platforms have become a pivotal focus by integrating nanomaterials with biomolecule detections such as metabolomics [17, 18, 19, 20] and peptideomics [21, 22]. The efficacy of a nano‐diagnostic platform is governed by two key elements: the precisely designed nanomaterials that can sensitively detect biomolecules with a simple operational protocol to ensure rapid diagnosis, and the clinical relevance of the detected biomolecules to the disease. Compared to peptideomics, glycomics are more closely related to disease processes as they significantly influence the stability and biological functionality of proteins [23, 24]. Furthermore, in comparison to downstream metabolomics, changes in glycomics usually occur earlier in the disease progression and offer greater potential for early disease detection [25, 26, 27, 28, 29]. However, despite the significance of the glycomics in disease development, they are scarcely incorporated in large‐scale clinical diagnosis due to their extensive structural heterogeneity, low abundance, and complicated pretreatment processes [30, 31, 32]. Therefore, well‐designed advanced nanomaterials are critical to overcome these limitations and bridge the gap between disease‐specific glycans and their high‐throughput diagnostic applications.

Graphitized carbon (GC) nanomaterials are the most commonly used for research on underivatized glycans owing to their capacity for hydrophobic effects, electron‐induced dipole interactions, and electronic repartition interactions with glycans [33, 34, 35, 36]. Nevertheless, due to the simple structure and morphology, traditional GC materials usually necessitate their coupling with chromatography for glycan detection. This prolongs analysis time and hinders their clinical‐scale application [37, 38, 39]. Previously, our group developed some porous GC materials based on metal–organic frameworks (MOFs) to decode N‐glycans in complex bio‐species and explored their disease diagnosis applications [40, 41, 42, 43]. Although these MOFs‐derived porous GC structures can provide rich surface morphology and enable chromatography‐free glycan research, their relatively small pore sizes (micro‐mesoporous degree) often limit mass transfer [44] for glycan adsorption and consequently compromise research efficiency. Accordingly, designing and constructing an advanced GC nanostructure that incorporates multi‐sized pore structures with interconnected expanded channels appears to be an effective solution for fully leveraging the three‐dimensional interpenetrating pore architectures to enhance glycan retention and improve mass transport efficiency. This precisely nanoarchitectural designer is expected to enhance the efficiency of glycan research, streamline the diagnostic workflow, and ultimately meet the demands of large‐scale, high‐throughput clinical disease diagnosis.

**FIGURE 1:**
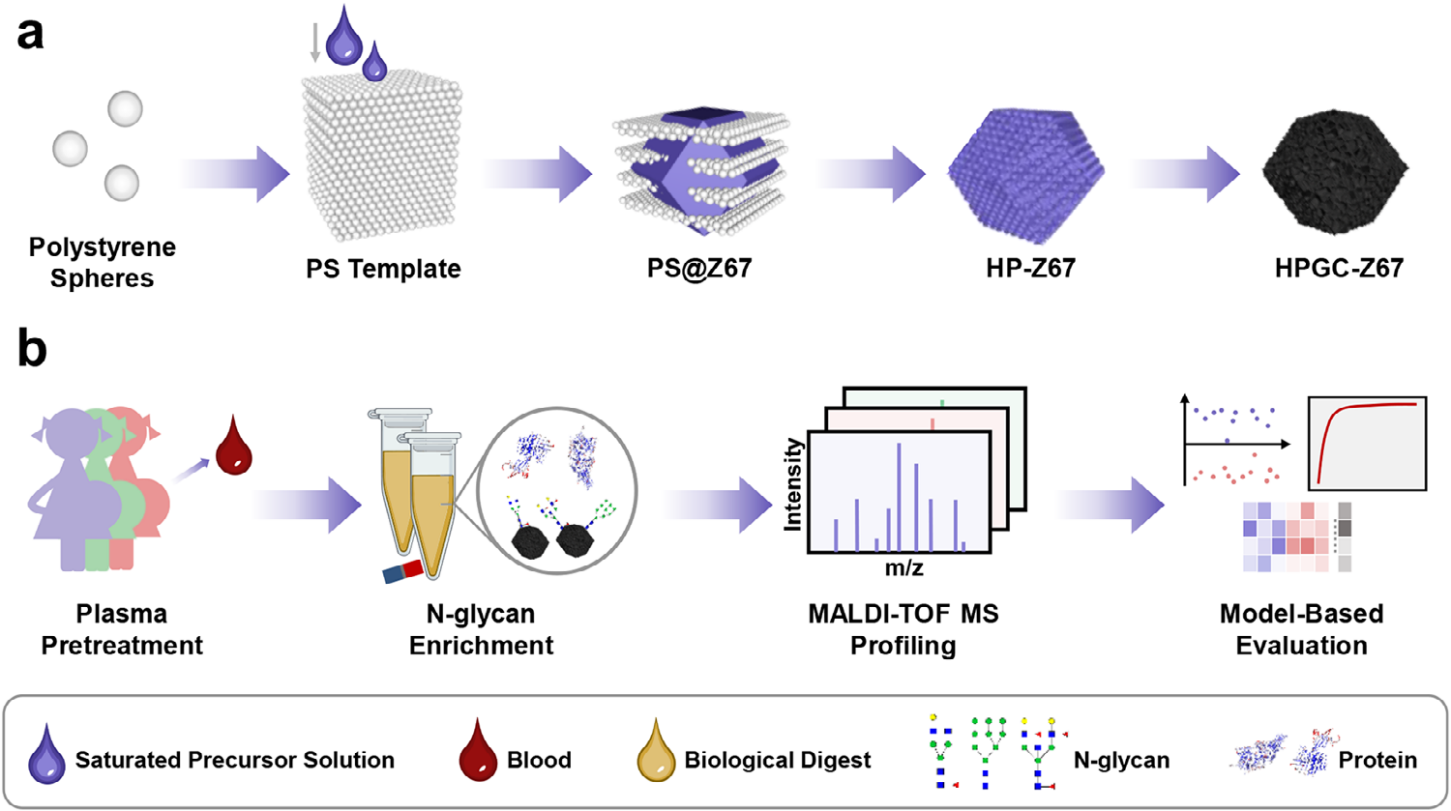
(a) Schematic illustration showing the construction of HPGC‐Z67 and (b) its diagnostic workflow for intrapartum fever.

Herein, we designed a hierarchical channeled graphitized carbon (HPGC‐Z67) through an integrated strategy combining hard templates, solvent‐controlled nucleation, and high‐temperature calcination (Figure 1). HPGC‐Z67 characterizes a high degree of graphitization and hierarchical interconnected channels, promoting efficient N‐glycan retention and intra‐channel diffusion. Thanks to the strong interaction sites provided by the 3D interpenetrating channels, the HPGC‐Z67 nano‐diagnostic platform can enhance glycan detection efficiency and simplify the workflow, thereby bridging the technological gap between disease‐specific glycan detection and high‐throughput diagnostic implementation. Combined with MALDI‐TOF MS, the HPGC‐Z67 platform allowed sensitive extraction of N‐glycan profiles from 150 plasma samples (health controls, ERMF, and CAM). Eight key N‐glycans features were finally screened from these N‐glycan profiles to realize the diagnosis of intrapartum maternal fever and stratification of ERMF and CAM. Notably, we also discovered two crucial N‐glycans that are sensitive to inflammatory infection fever (IF‐sensitive) and non‐infection fever (n‐IF‐sensitive), respectively. Their combination can simultaneously differentiate no fever health controls, ERMF, and CAM with an area under the curve (AUC) of 0.965 in the training set and 0.914 in the validation set. Our HPGC‐Z67 nano‐diagnostic platform holds potential for large‐scale clinical glycomics, promising enhanced biomarker discovery and early disease detection, with applications extending beyond intrapartum fever.

## 2 Results and Discussion

### 2.1 Construction and Characterization of HPGC‐Z67

**FIGURE 2:**
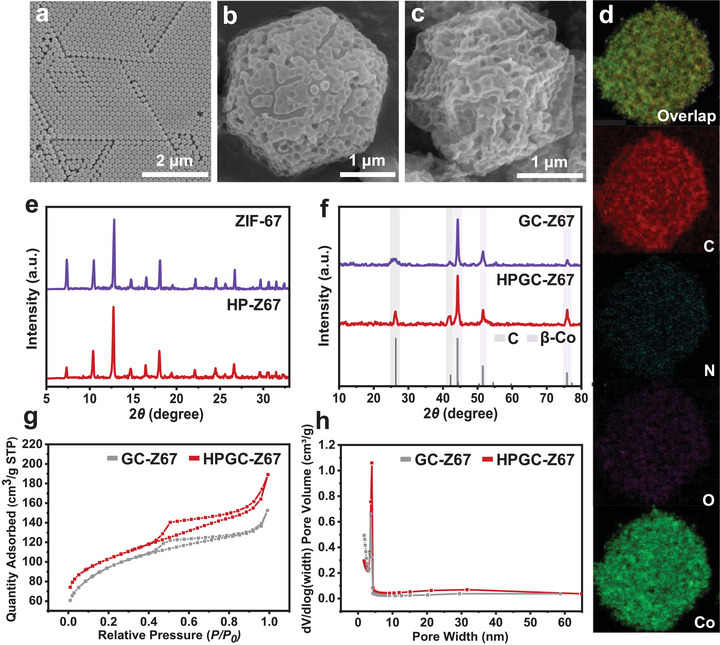
Characterization of HPGC‐Z67, HP‐Z67, GC‐Z67, and ZIF‐67. SEM images of (a) PS template, (b) HP‐Z67, and (c) HPGC‐Z67. (d) Overlapped image and corresponding element mapping images of HPGC‐Z67. (e) Powder XRD patterns of ZIF‐67 and HP‐Z67. (f) Powder XRD patterns of GC‐Z67 and HPGC‐Z67 and their corresponding standard patterns. (g) Nitrogen adsorption/desorption isotherms and (h) pore size distribution curve of GC‐Z67 and HPGC‐Z67.

The HPGC‐Z67 is designed and constructed according to the procedure presented in Figure 1a, which consists of three main steps: preparation of hard templates, construction of hierarchical porous MOF (HP‐Z67), and high‐temperature graphitization. Firstly, the prepared monodisperse polystyrene spheres (PSs) were self‐assembled into a three‐dimensional ordered PS template by centrifugation. The scanning electron microscope (SEM) image in Figure 2a demonstrates that the prepared PSs are closely and orderly arranged in a hexagonal close‐packed structure with a diameter of 163.7 ± 5.3 nm (*n* = 10). Subsequently, the saturated precursor (2‐methylimidazole and Co(NO_3_)_2_·6H_2_O) solution was filled into the gaps of the PS template by negative pressure to ensure that the nucleation process of HP‐Z67 occurred inside the PS template [45, 46]. Then, the HP‐Z67 nucleated and grew in a double‐solvent system and obtained a hierarchical porous structure after removing the PS template [47, 48]. As shown in Figure 2b, HP‐Z67 presents a porous hexagonal prism structure with an average pore diameter of 162.8 ± 8.7 nm, which coincides with the PS size, indicating the successful construction of porous structures according to the PS template. At last, high‐temperature treatment under N_2_ atmosphere was conducted to co‐construct HP‐Z67‐based hierarchical porous channeled, highly graphitized, magnetic carbon materials, namely HPGC‐Z67. Figure 2c shows that HPGC‐Z67 still retains the crystal morphology and macroporous structure of HP‐Z67, demonstrating its structural thermal stability. These macropores are interconnected to form channels, which promote the diffusion and binding of N‐glycans within HPGC‐Z67 architectures. Moreover, the overall size of HPGC‐Z67 is 2407.9 ± 114.1 nm, which is reduced compared with 2821.2 ± 104.9 nm of HP‐Z67, attributed to the volume shrinkage caused by the conversion of organic ligands to graphitized carbon at high temperature. For comparison, we synthesized regular ZIF‐67 with classical rhombic dodecahedral structures with an average size of 1186.3±40.5 nm (Figure S1a,b). Similarly, graphitization was also performed on ZIF‐67 to yield the graphitic carbonized ZIF‐67 (GC‐Z67) with a size of 814.4±50.8 nm (Figure S1c). Notably, compared with ZIF‐67 and GC‐Z67 (Figure S2a,b), the uniformly distributed macroporous structure can be clearly observed from the transmission electron microscopy (TEM) images of both pristine HP‐Z67 and calcinated HPGC‐Z67 (Figure S2c,d), suggesting the successful construction of hierarchical porous structures with expanded channels. The element mapping images of HPGC‐Z67 in Figure 2d reveal the even distribution of Co, C, N, and O in HPGC‐Z67 with corresponding atomic ratios of 17.10%, 76.35%, 3.35% and 3.20%, respectively. Such a high C concentration suggests sufficient affinity sites for N‐glycan recognition through C‐C interaction.

A higher degree of graphitization can enhance the interaction between the nanomaterial and the N‐glycans, improving enrichment efficiency [49]. To investigate the graphitization of the carbon in HPGC‐Z67, we examined the crystallographic structures using X‐ray diffraction (XRD) patterns. Compared to the XRD patterns of the pristine MOF of HP‐Z67 (Figure 2e), two distinct diffraction peaks of graphitized carbon can be detected at 26.5° and 42.3°, which correspond to (002) and (100) lattice planes of graphitized carbon (PDF#41‐1487) (Figure 2f), demonstrating the successful graphitization of HPGC‐Z67. Meanwhile, Co exists as β‐Co (PDF#15‐0806) of which the (111), (200), and (220) lattice planes are observed at diffraction peaks at 44.2°, 51.5°, and 75.8°. Moreover, the XRD patterns of HPGC‐Z67 and HP‐Z67 are consistent with those of their microporous counterparts, GC‐Z67 and ZIF‐67, indicating that well‐crystallized structures are maintained during the hierarchical porous construction process. Also, the D peak (1350 cm^−1^) and G peak (1589 cm^−1^) in the Raman spectra further verify the existence of graphitized carbon in HPGC‐Z67 and GC‐Z67 (Figure S3a,b). Notably, the I_D_/I_G_ of HPGC‐Z67 is 0.91, which is lower than that of GC‐Z67 (0.96). This indicates that HPGC‐Z67 has a higher degree of graphitization compared to GC‐Z67, which is more conducive to the retention of N‐glycans. Furthermore, we measured the pore structures of HP‐Z67 and HPGC‐Z67 via N_2_ adsorption/desorption isotherms and pore size distribution testing. As expected, HP‐Z67 shows a hierarchical porous structure with Type I and Type IV isotherms, indicating micropores and mesopores (Figure S4a). The average pore diameters are 1.3 nm and 4.0 nm (4 V/A by Brunauer−Emmett−Teller (BET)), respectively (Figure S4b). Comparatively, the ZIF‐67 only exhibits a Type I isotherm feature indicative of micropores with the average pore diameter of 1.7 nm (Figure S4c,d). After graphitization, the HPGC‐Z67 retains the mesoporous structure from its pristine MOF and exhibits Type IV isotherm characteristics with a larger pore size of 4.0 nm than that of 3.7 nm for GC‐Z67 (Figures 2g,h). This hierarchical porous structure benefits N‐glycan diffusion across interconnected porous channels, while also blocking large protein interferents and enhancing the specificity of N‐glycan recognition. Remarkably, HPGC‐Z67 has a BET surface area of 371.4 m^2^ g^−1^ and a total pore volume of 0.28 cm^3^ g^−1^, overwhelming those of GC‐Z67, which are 320.1 m^2^ g^−1^ and 0.24 cm^3^ g^−1^, respectively. The larger specific surface area and pore volume of HPGC‐Z67 may be attributed to the hierarchical porous structure preserved after graphitization. This favorable surface area can provide sufficient retention sites for N‐glycan retention.

### 2.2 Detection Performance of the HPGC‐Z67 Platform for N‐glycans

**FIGURE 3:**
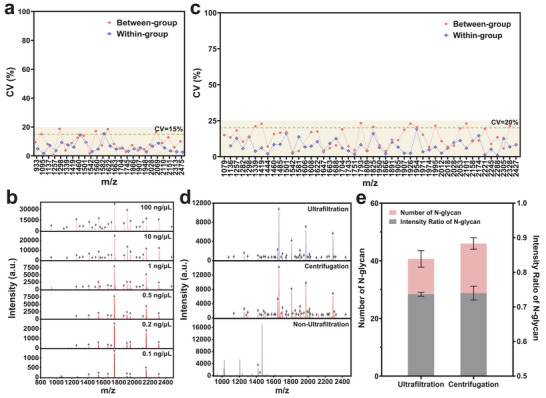
Evaluation of the HPGC‐Z67 performance for N‐glycan detection. (a) The CV values for N‐glycan signals enriched and detected from OVA digest in between‐group and within‐group parallel tests (*n* = 3). (b) MALDI‐TOF MS spectra of N‐glycans enriched and detected from OVA digest at different concentrations. (c) The CVs for N‐glycan signals enriched from plasma digest in between‐group and within‐group parallel tests (*n* = 3). (d) MALDI‐TOF MS spectra of N‐glycans enriched and detected from plasma digest by non‐ultrafiltration, ultrafiltration, and centrifugation methods. (e) The number of N‐glycans enriched and their intensity ratio relative to the total signal across parallel tests (*n* = 3) by the ultrafiltration and centrifugation method.

Following the process displayed in Figure 1, we conducted a preliminary evaluation of the N‐glycan detection performance of the HPGC‐Z67 platform using OVA digest as a glycan standard. Specifically, HPGC‐Z67 was introduced into a 1 µg µL^−1^ OVA digest to capture the glycans, which were then detected by MALDI‐TOF MS. Firstly, the reproducibility of the HPGC‐Z67 platform for standard OVA was investigated through three between‐group parallel experiments, as well as three within‐group parallel signal collections. The results in Figures S5 and S6 show that HPGC‐Z67 enriched 25 OVA‐related N‐glycans (Table S1) with consistent signal intensity in both between‐group and within‐group tests. The coefficients of variation (CVs) vary from 0.7% to 18.7% between groups (average CV = 9.2%), and from 1.7% to 15.4% within groups (average CV = 6.0%) (Figure 3a). This robust reproducibility demonstrates a stable technical foundation to yield reliable results in subsequent experiments.

The retention mechanisms of porous graphitized carbon (PGC) materials are complex and have been extensively discussed, but they are not conclusively proven yet [36, 49]. In glycomics, the affinities of PGC for glycans primarily involve electron‐induced dipole interactions, electron redistribution interactions, and hydrophobic effects [49]. Since most glycans are hydrophilic and polar, they are retained through charge induction on the polarizable PGC surface [50]. For a small number of hydrophobic glycans (such as phosphatidylinositol anchors), they may also be enriched on the graphitized carbon through hydrophobic interactions [51]. Moreover, PGC is electron‐rich, and the retention performance can be enhanced by electrostatic interactions. These π‐electron interactions intensify with an elevated degree of graphitization [52]. Notably, most traditional graphite materials have planar structures and tend to adsorb planar analytes more effectively, as their parallel arrangement enhances proximity between molecules and the material surface [36]. Inspired by this, considering that glycans are not planar molecules but possess complex three‐dimensional conformations, PGC materials with three‐dimensional porous structures are expected to better match the spatial configurations of target glycans and thus improve glycan retention compared to planar graphitic materials. In particular, PGC materials with a designed hierarchical interconnected porous structure, such as HPGC‐Z67, are anticipated to achieve higher retention efficiency because their multi‐scale pore channels can accommodate glycans with varying degrees of polymerization. To validate this hypothesis, we evaluated the feasibility of N‐glycan analysis utilizing HPGC‐Z67, employing HP‐Z67, ZIF‐67, and GC‐Z67 as comparisons (Figure S7a,b). The results show that neither pristine HP‐Z67 nor ZIF‐67 can effectively analyze N‐glycans as MS spectra obtained using them are cluttered with plenty of interference signals, and no N‐glycan can be recognized. Notably, both graphitized HPGC‐Z67 and GC‐Z67 demonstrate significant N‐glycan detection capability, yielding clear N‐glycan signals. As expected, owing to its higher graphitization degree and hierarchical interconnected porous structure, HPGC‐Z67 produces signals with higher intensity than GC‐Z67.

Furthermore, we compared the enrichment and detection performance of HPGC‐Z67 and GC‐Z67 for low‐concentration N‐glycans using a series of diluted OVA digest (100, 10, 1, 0.5, 0.2, 0.1 ng µL^−1^). As seen in Figure 3b and Figure S8, HPGC‐Z67 outperformed GC‐Z67 in terms of the number of enriched N‐glycans and their signal intensities. Especially, even at an ultra‐low concentration of 0.1 ng µL^−1^, HPGC‐Z67 was still able to detect 8 N‐glycans (S/N > 3), whereas GC‐Z67 only detected 2. This impressive analysis performance of HPGC‐Z67 surpassed a series of previously reported carbon materials [32, 40, 41, 42, 43, 44, 53, 54]. It may be due to the higher graphitization of carbon, which improves interaction with N‐glycans, and a larger surface area that offers abundant adsorption sites for N‐glycans, as well as hierarchically interconnected porous that facilitate the diffusion and retention of N‐glycans inside the channels. Given the hierarchical channeled structure of HPGC‐Z67 has potential for blocking large interferences through size exclusion effect, we explore its detection feasibility for more complex samples, where high concentration proteins are usually enriched by non‐specific adsorption and inhibit N‐glycan signals via ion suppression in the MS process [32, 55, 56]. OVA digests were mixed with OVA and bovine serum albumin (BSA) at the mass ratio of 1:500:500 (OVA digest: OVA: BSA, m/m/m) to yield the model sample. The results show that N‐glycans are undetectable before treatment with HPGC‐Z67 (Figure S9a) due to the existence of large amounts of proteins (Figure S9b). Interestingly, after treatment with the HPGC‐Z67 platform, 22 N‐glycan signals are detected (Figure S9c) while the protein signals are nearly obscured by the baseline (Figure S9d). These findings confirm effective capture and detection of N‐glycans by the HPGC‐Z67 platform from protein‐rich surroundings via the size exclusion effect, thereby validating our strategy of sensitive and specific N‐glycopeptide detection by designing graphitized material with hierarchical channeled structures. Further comparison of the capture performance of HPGC‐Z67 with that of reported N‐glycan recognition materials (Table S2) demonstrates the superiority of our HPGC‐Z67‐based protocol.

For large‐scale clinic applications, simplicity, rapidity, and ease of operation are crucial, as they directly determine diagnostic efficiency. Plasma digestion to release N‐glycans from glycosylated proteins using PNGase‐F enzyme is essential for N‐glycans profiling. However, this process involves ultrafiltration steps to remove reducing/alkylating reagents in the plasma digests, which is time‐consuming and costly. Herein, we attempted to improve the N‐glycan release protocol based on the HPGC‐Z67 platform. Before this, considering the stability of the method may be perturbed by the complexity of real samples, we assessed the robustness of N‐glycan enrichment and detection in plasma digest mixtures using the HPGC‐Z67 platform to ensure the reliability of protocol optimization. As a result, consistent intensities (Figure S10a,b) are observed between N‐glycan signals detected in the between‐group (CVs range from 3.0% to 23.3%, mean CV = 13.2%) and within‐group (CVs range from 0.9% to 19.2%, mean CV = 7.5%) across three parallel tests (Figure 3c). These results reveal that the HPGC‐Z67 platform maintains robust technical reliability even when applied to more complex biological samples. Encouraged by this, we replaced the conventional ultrafiltration procedure with direct centrifugation and washing. After the reduction and alkylation reaction, the plasma solution with reducing/alkylating reagents is centrifuged for 2 min, and the supernatant is discarded. The protein precipitate is then washed three times with 200 µL of ABC buffer to completely remove residual reagents. Compared to the traditional ultrafiltration process, our HPGC‐Z67 platform‐based protocol significantly decreases centrifugation time (from 32 min to only 6 min) and reduces the cost of one ultrafiltration tube (approximately CNY 30) per plasma sample. More importantly, the analysis results in Figure 3d demonstrate that our direct centrifugation protocol overwhelms both the non‐ultrafiltration assay and conventional ultrafiltration in terms of the number and intensity ratio of enriched N‐glycans (Figure 3e; Table S3). The effectiveness of our platform is attributed to its effectiveness for the capture and detection of N‐glycans, even when not thoroughly removing residual reagent interference. Finally, to achieve optimal enrichment and detection performance, we carefully evaluated the effects of the N‐glycan to materials ratio, incubation time, and elution time on the analysis efficiency. As shown in Figure S11a,b, HPGC‐Z67 achieves its highest detection efficiency for plasma N‐glycans at the ratio of 15 µL plasma digest: 20 µL materials (10 µg µL^−1^), and demonstrates superior performance over GC‐Z67 across all tested ratios. Additionally, an incubation and elution time of 60 min was proven to be the optimal conditions since binding saturation occurs without increases in the number and intensity ratio of N‐glycans as time prolongs. (Figure S12a,b). Overall, the HPGC‐Z67‐based N‐glycan detection platform features high reliability, time‐effectiveness, and low cost in plasma analysis, which is beneficial for large‐scale clinical applications.

### 2.3 Diagnostic Model Optimization of the HPGC‐Z67 Platform

**FIGURE 4:**
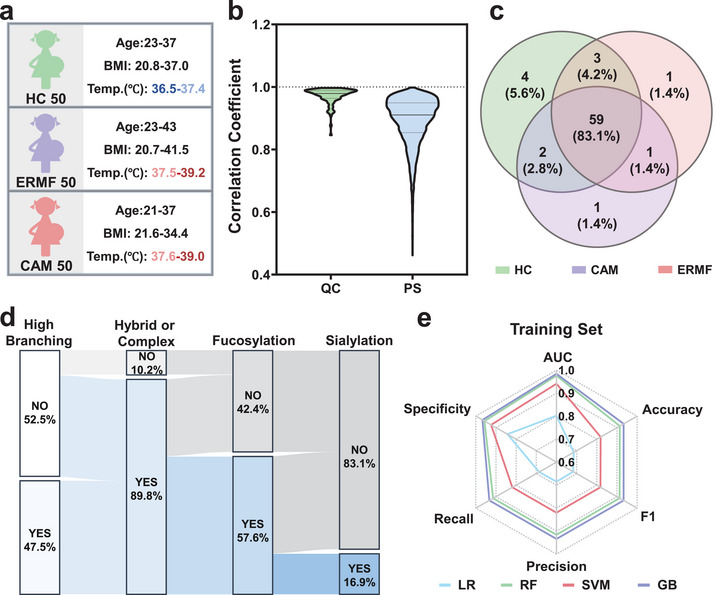
Evaluation of machine learning model classification performance across three cohorts. (a) Sample information for the three cohorts (*n* = 150), including HC, ERMF, and CAM. (b) Distribution of Pearson correlation coefficients within QC samples and all plasma samples. (c) Venn diagram representing the number of N‐glycans detected across the three cohorts. (d) Sankey diagram representing the distribution of 59 N‐glycan types detected across all three cohorts. (e) Radar chart of comprehensive evaluation metrics for four machine learning models predicting three cohorts.

With the simplified process protocol and optimized detection conditions, we extracted N‐glycan profiles from 150 plasma samples using the HPGC‐Z67 platform following the workflow illustrated in Figure 1b. All the plasma samples were collected from pregnant women prior to delivery and underwent clinical diagnosis. As shown in Figure 4a, the participants include healthy controls (HC, n = 50) without analgesia and no fever symptoms (< 37.5°C), the ERMF group (n = 50) received analgesia and had non‐infectious fever (≥ 37.5°C), and the CAM group (*n* = 50) received analgesia and had chorioamnionitis‐infectious fever (≥ 37.5°C). More detailed information about the plasma samples is provided in Table S4. MS spectra of all these plasmas were processed using the MALDIquant package in RStudio, as described in the Statistical Analysis section.

To ensure the upcoming screening of N‐glycan features and establishing the predictive diagnostic model are not impacted by batch variations, we mixed six plasma digests as a quality control (QC) group and conducted 15 sample processing and detection. The CVs assessment across 15 experiments yields satisfying results of below 10% for both the number and intensity ratio (Figure S13). Further principal component analysis (PCA) in Figure S14 reveals a tighter clustering of QC samples than all plasma samples, confirming the effective control of batch variations. Moreover, strong correlations are observed among QC samples with Pearson correlation coefficients exceeding 0.846 (Figure 4b). In contrast, plasma samples exhibited a broader range of correlation coefficients (0.462‐0.999). These results indicate that the technical variability during experiments is substantially smaller than the biological inter‐sample variation, demonstrating the reliability of subsequent clinic symptom‐related plasma N‐glycan analysis.

We applied the HPGC‐Z67 platform in plasma samples and identified a total of 71 N‐glycans (Table S3) in three plasma cohorts (*n* = 150), of which 59 N‐glycans were identified in all three groups, accounting for 83.1% (Figure 4c). A total of 89.8% of these 59 N‐glycans are hybrid or complex glycosylation types (with 57.6% of fucosylation and 16.9% of sialylation), while the remaining 10.2% are high‐mannose type (Figure 4d). Additionally, the 59 N‐glycans show highly branched characteristics, with 47.5% containing more than four N‐acetylglucosamine (GlcNAc) residues. An overview of the signal intensities of these 59 N‐glycans in each cohort is presented as a heatmap in Figure S15a. To prevent randomness and ensure the reliability of the predictive diagnostic model, the subsequent machine learning was based on these 59 N‐glycans. PCA analysis was initially used to differentiate the N‐glycans levels across different cohorts (Figure S15b). However, direct classification based on raw mass spectrometry data proved challenging due to the inherent limitations of the linear dimensionality reduction of PCA in capturing complex nonlinear patterns. Therefore, we introduced more machine learning models capable of handling nonlinear modeling and enabling modeling and optimization in high‐dimensional feature space to capture subtle classification patterns embedded within complex mass spectrometry data. These models included logistic regression (LR), support vector machine (SVM), random forest (RF), and gradient boosting (GB). 150 samples were randomly divided into training and validation sets at a ratio of 7:3, with each set consisting of HC, ERMF, and CAM in equal proportions. Then, we evaluated the classification performance of the above four machine learning algorithms on the training and validation sets based on plasma N‐glycan profiles. Meanwhile, stratified tenfold cross‐validation was adopted to improve the generalization ability and data utilization of models, reducing the overfitting with the limited data scale [57, 58]. The receiver operating characteristic (ROC) curves for all the models are shown in Figure S16a,b. The area under the curve (AUC) values of SVM, RF, and GB exceed 0.9 in both the training and the validation sets, significantly higher than those of LR. A comprehensive evaluation of each algorithm was conducted using radar charts incorporating metrics including AUC, classification accuracy, F1 score, precision, specificity, and recall (Figure 4e; Figure S16c). Overall, RF and GB outperformed SVM and LR, with GB achieving the best results, suggesting the screening potential of the GB model for the three cohorts based on N‐glycan profiles. Detailed values of all evaluation metrics are provided in Table S5.

Using the optimized model, the multivariate classification of HC, ERMF, and CAM cohorts was conducted with a one‐vs‐rest strategy based on N‐glycans extracted by the HPGC‐Z67 platform (Figure S17a,b). High AUC values were achieved for each cohort, ranging from 0.988 to 0.995 in the training set and from 0.953 to 0.993 in the validation set, confirming its excellent classification capability. The 3 × 3 confusion matrices are presented in Figure S17c,d, with specific metrics detailed in Table S6. Notably, all cohorts achieved accuracies exceeding 91.1% and recall rates above 91.4% in both the training and validation sets, except the HC cohorts had a slightly lower recall of 80.0% in the validation set. These results demonstrate the precise prediction ability of the HPGC‐Z67 platform with the GB model, especially for ERMF and CAM. Further pairwise discrimination was performed among the HC, ERMF, and CAM groups. The sample‐level plots in Figure S18a–f demonstrate the ability of the platform to accurately categorize the majority of the samples. The ROC curves confirmed the robustness of the GB model for pairwise typing across the three cohorts, showing high AUC values in both training (HC/CAM: 0.973, HC/ERMF: 0.991, and CAM/ERMF: 0.993) and validation (HC/CAM: 0.973, HC/ERMF: 1.000, and CAM/ERMF: 1.000) sets (Figure S19a,b). It is noteworthy to mention that even when applied to the clinically challenging CAM and ERMF groups, the extracted N‐glycan profiles demonstrated robust discriminatory power with all metrics (AUC, accuracy, recall, and specificity) exceeding 94.3% in both training and validation sets (Table S7). These results proved that plasma N‐glycans extracted by the HPGC‐Z67 platform enable rapid and precise early warning and diagnosis of CAM and ERMF, facilitating proactive measures for delivery and postpartum recovery.

### 2.4 Precise Intrapartum Fever Diagnosis by the HPGC‐Z67 Diagnostic Platform

**FIGURE 5:**
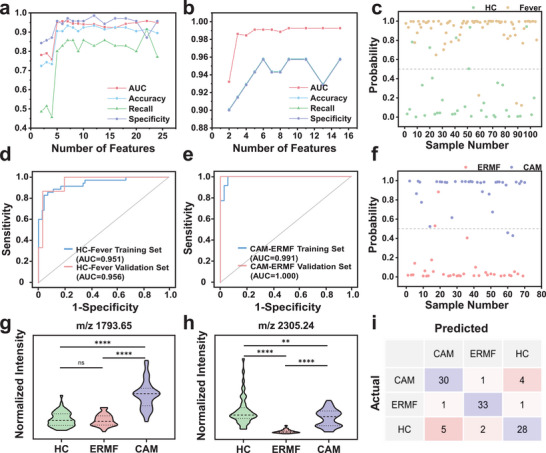
Screening of key feature sets associated with intrapartum fever. N‐glycan screening based on model evaluation metrics and feature importance scores in the training set of (a) HC/Fever and (b) CAM/ERMF. The sample‐level plot depicting the probability predicted by screened key N‐glycans in the training sets for differentiating (c) HC/Fever and (d) CAM/ERMF. The ROC curves for classifying (e) HC/Fever and (f) CAM/ERMF based on key N‐glycans across training and validation sets. (g, h) Violin plot analysis of Feature Set 3 in HC/CAM/ERMF. ns, *p* > 0.05; ^*^, *p* < 0.05; ^**^, *p* < 0.01; ^***^, *p* < 0.001; ^****^, *p* < 0.0001. (i) The confusion matrix for predicting HC/CAM/ERMF based on Feature Set 3 in the training set.

Motivated by the high classification efficacy demonstrated by the 59 N‐glycan features extracted by the HPGC‐Z67, we are committed to identifying the key N‐glycans most closely associated with fever during labor, particularly those critical for distinguishing CAM‐induced infectious fever that requires prompt prenatal intervention. Based on the classification results, we ranked the importance of 59 N‐glycan features using the Feature Importance Score and a *p*‐value < 0.05 as screening criteria in the HC/fever groups (a mixture of CAM and ERMF) and the CAM/ERMF groups, respectively. Subsequently, we progressively expanded the feature sets according to their importance and approached the classification prediction. The performance of the feature sets is comprehensively evaluated using metrics including AUC, accuracy, recall, and specificity. As shown in Figure 5a,b and Figure S20a,b, generally, incorporating more N‐glycan features into the classification model improves diagnostic performance, but raises the complexity of data collection. On the other hand, using too few features undermines the discriminatory power of the prediction model. Ultimately, five key N‐glycan features (m/z 1688.61, m/z 1751.60, m/z 1793.65, m/z 1866.66, and m/z 2305.24) were screened for the classification of the HC/Fever groups and denoted as Feature Set 1. Another five key N‐glycan features (m/z 1282.45, m/z 1745.63, m/z 1793.65, m/z 1809.64, m/z 2305.24) were selected as the Feature Set 2 to discriminate the CAM/ERMF groups. Detailed information about these two feature sets is provided in Table S8. All these key N‐glycans exhibited hybrid or complex types, with 62.5% displaying fucosylation, 12.5% containing sialic acid, and 50% presenting highly branched structures. As displayed in Figure 5c,d and Figure S21a, Feature Set 1 achieves successful classification for the HC/Fever groups in both training (AUC = 0.951, accuracy = 90.5%, specificity = 95.7%) and validation (AUC = 0.956, accuracy = 93.3%, specificity = 96.7%) sets (Table S9). This good differentiation performance verifies the capability of the selected key N‐glycan features in discriminating between fever individuals during delivery from non‐fever samples. Further classification of CAM/ ERMF groups by the screened Feature Set 2 also obtained significant discriminatory performance in the training set, with an AUC of 0.991, accuracy of 94.3%, and specificity of 94.3% (Figure 5e,f; Figure Table S10). Especially, they even achieved 100% of accuracy and specificity on the validation set (Figure S21b; Table S10). This accurate prediction of CAM/ERMF enables timely and precise fever alerts with different etiologies for pregnant women prior to surgery.

Subsequently, expression levels of these screened key N‐Glycans are explored and displayed as violin plots. Among the key N‐glycan features for HC/Fever prediction, features m/z 1688.61, m/z 1751.60, m/z 1793.65, and m/z 1866.66 are upregulated, while the feature of m/z 2305.24 is significantly downregulated in the intrapartum fever group compared to the HC group (Figure S22). Further comparison of Key N‐Glycan features for fever subdivision in Figure S23 reveals that all feature m/z 1282.45, m/z 1745.63, m/z 1793.65, m/z 1809.64, and m/z 2305.24 are upregulated in the CAM group relative to non‐infectious ERMF. Moreover, the Glycan type of these key features reveals that most plasma fucosylation and sialylation N‐glycans levels are increased in the fever group compared to the non‐fever group, except for the fucosylation N‐glycan of m/z 2305.24. Remarkably, 80% of the up‐regulated key features in CAM are fucosylation N‐glycans, highlighting the cohesion relationship of fucosylationin with maternal fever, including both infection‐induced and analgesia‐induced fevers. This relationship is supported by reported studies, which have shown elevated expression of antennary fucosylated structures during autoimmune or inflammatory processes [59, 60]. Also, terminally fucosylated hemopexin is observed to be elevated in CAM‐induced bacterial inflammation. This protein activates macrophages to release cytokines such as IL‐6 and IL‐8 [7, 61, 62, 63], which act on the hypothalamus to induce fever and uterine contractions [64, 65, 66, 67]. Interestingly, two key fucosylation N‐glycan features of m/z 1793.65 (H4N4F2) and m/z 2305.24 (H5N5F3) are overlapping screened in both HC/Fever and CAM/ERMF classification models. We speculated that these two N‐glycans have the potential to serve as key indicators for distinguishing among the HC/CAM/ERMF cohorts and therefore further assessed their expression levels across these three cohorts. As shown in the violin plot of Figure 5g, the N‐glycan at m/z 1793.65 exhibits no noticeable alteration between HC and ERMF, but marked up‐regulation in CAM compared to HC and ERMF, suggesting a strong association with inflammatory infection fever (IF‐sensitive). Meanwhile, the key N‐glycan feature of m/z 2305.24 demonstrates apparent downregulation in ERMF relative to both HC and CAM (Figure 5h), indicating its cohesive connection with non‐infection fever (n‐IF‐sensitive). These two features are assigned as Feature Set 3, and their expression across the three cohorts is visualized in the heatmap of Figure S24a. Surprisingly, Feature Set 3 distinguishes HC/CAM/ERMF groups simultaneously with an excellent prediction performance (Figure 5i; Figure S24b). The training and validation sets achieve AUC values of 0.965 and 0.914, accuracy rates of 86.7% and 80.0%, and specificities of 93.3% and 90.0%, respectively (Table S11). These findings validated that the IF‐sensitive N‐glycan features and n‐IF‐sensitive N‐glycan features are highly effective for the simultaneous diagnostic prediction of HC, CAM, and ERMF. Given the high specificity of these two screened N‐glycans extracted by the HPGC‐Z67 platform, they may hold great potential as crucial biomarkers of maternal fever for more clinical applications.

## 3 Conclusion

In this work, we designed a hierarchical channeled graphitized carbon (HPGC‐Z67) as a rapid and sensitive nano‐diagnostic platform. With carefully nano‐architectural designing, HPGC‐Z67 features a high surface area and high graphitization degree, together with interconnected hierarchical channels. These advantages allow the HPGC‐Z67 platform to enhance N‐glycan retention and diffusion within hierarchical channels. Additionally, leveraging the sensitive glycan detection capability, the HPGC‐Z67 nano diagnostic platform enables the streamlining of experimental procedures, reducing processing time by approximately 25 min and cutting costs by about CNY 30 per sample compared to standard methods. The HPGC‐Z67 nano‐diagnostic platform demonstrated robust capabilities for high‐throughput and reproducible N‐glycan profiling from 150 clinical plasma samples. Combined with machine learning, we screened two feature sets comprising eight key N‐glycans from the acquired glycan profiles. These key N‐glycans enabled accurate discrimination not only between fever and healthy states but also between infectious (CAM) and non‐infectious (ERMF) etiologies, with all AUC values exceeding 0.95 in training and validation cohorts. Notably, a model based on just two potential biomarkers, an IF‐sensitive and an n‐IF‐sensitive N‐glycan, achieved simultaneous differentiation of HC, CAM, and ERMF with high AUC values (0.965 in training, 0.914 in validation). Our HPGC‐Z67 nano‐diagnostic platform holds the potential for the precise and early diagnosis of intrapartum fever and timely clinical interventions, expanding the scope of nano‐diagnostic technologies to bio‐detection of glycomics.

## 4 Experimental Methods

### 4.1 Synthesis of 3D Ordered PS Templates

Given previous reports [46], 70 mL styrene was repeatedly washed with 20 mL NaOH aqueous solution (10 wt%) and water, respectively. Afterward, 65 mL of styrene was poured into 500 mL of water, and 2.5 g of PVP was then added. After passing nitrogen into the mixed solution for 10 min, the mixture was stirred continuously at 75°C for 0.5 h. Next, 1 g of K_2_S_2_O_8_ was dissolved in 50 mL of water and rapidly poured into the above mixed system, then kept at 75°C with stable stirring for 24 h. The white product was cooled down to ambient temperature and kept centrifuged at 4000 rpm for 12 h. Subsequently, white precipitate was obtained and vacuum‐dried overnight at 50°C to yield 3D ordered polystyrene sphere templates.

### 4.2 Synthesis of HP‐Z67 and HPGC‐Z67

Inspired by previous reports [48], Co(NO_3_)_2_·6H_2_O and 2‐methylimidazole were dissolved in methanol to make the final concentrations of 0.4 g mL^−1^and 1 g mL^−1^, respectively. The PS templates were immersed in 20 mL of the above solution for 10 min, then transferred to a vacuum oven for another 45 min to fill the pores of the PS templates with the reaction solution. The immersed PS templates were transferred to clean beakers and vacuum dried at 45°C overnight. After that, purple PS templates were obtained and immersed in 30 mL of a mixed solution of methanol and ammonia with equal volumes. 2 pieces of weighing paper were wrapped around the mouth of the beaker, and then degassed under vacuum for 15 min. During this process, the growth of the ZIF‐67 leads to increased pressure and splits the PS templates into fragments. After soaking the mixed solution overnight at room temperature, the PS fragments were extracted and dried under vacuum at 50°C. The obtained precipitates were washed with N,N‐Dimethylformamide (DMF) to elute the PS template, followed by washing with dichloromethane to remove the residual DMF. HP‐Z67 was obtained after vacuum drying at 50°C. The HP‐Z67 powder was put into a crucible and carbonized at 900°C for 1.5 h in a nitrogen atmosphere to prepare HPGC‐Z67.

### 4.3 Preparation of Plasma Digest

Plasma samples were removed from −80°C and thawed in 4°C environment. 15 µL of plasma sample was extracted, and the volume was fixed to 50 µL with 25 mm ABC buffer solution. The solution was incubated at 100°C for 15 min to induce protein denaturation and then cooled to room temperature. 2 µL of a mixture containing 500 mm tris(2‐carboxyethyl) phosphine and 1 mol L^−1^ 2‐chloroacetamide was added to the denatured protein solution. The reduction and alkylation reaction was carried out simultaneously for 30 min at room temperature and in the dark. The supernatant was separated by simple centrifugation for 2 min, and the remaining precipitate was remixed with 200 µL ABC buffer. The above washing operation was repeated three times, and the volume was finally fixed to 200 µL. As a control, the same protein solution was loaded into an activated ultrafiltration tube (3K, 0.5 mL), fixed to 400 µL with 25 mmol µL^−1^ABC buffer and centrifuged for 10 min at 14000 × *g*. The centrifuged solution in the ultrafiltration tube was subsequently refixed to 400 µL, centrifuged, and repeated three times. The remaining protein solution in the ultrafiltration tube was extracted, and the final volume was fixed to 200 µL. Finally, 1 µL of PNGase F enzyme was added to the protein solution and incubated at 37°C and 800 rpm. All plasma digests obtained were preserved at −20°C for subsequent use.

### 4.4 Enrichment of N‐Glycans from Biological Samples

A total of 20 µL of material suspension (10 µg µL^−1^) was thoroughly mixed with biological samples (including OVA digest, plasma digest, and mixtures of specific mass ratios of OVA, BSA, and OVA digests, etc.) and immobilized to 100 µL with 25 mm ABC buffer. The mixed solution was incubated at 37°C and 1200 rpm, followed by three washes with 100 µL of 25 mm ABC buffer for magnetic separation. 10 µL of 50% ACN solution was added, and the N‐glycan was eluted at 37°C and 800 rpm. The eluate was extracted by magnetic separation and then analyzed by MALDI‐TOF MS. The control experiments were performed as the above process by using different volumes of plasma digest (10 µL, 15 µL, 20 µL, 30 µL, 40 µL), different enrichment time (1 min, 5 min, 15 min, 30 min, 45 min, 60 min, 90 min) and elution time (1 min, 5 min, 15 min, 30 min, 45 min, 60 min, 90 min).

### 4.5 Statistical Analysis

The raw profiles (n = 3) of each sample from MALDI‐TOF MS were exported in.txt format by Bruker Compass flexAnalysis 3.4. The peak extraction, alignment, average, and normalization were conducted by MALDIquant package on RStudio to obtain a table containing m/z features and peak intensities [68]. In RStudio preprocessing, the signal‐to‐noise (SNR) and half‐window size were set to 3 and 120, respectively. The final N‐glycan output for the three cohort samples was obtained and screened for N‐glycan‐related m/z features in conjunction with the database in GlycoWorkbench software for subsequent analysis. The potential N‐glycan compositions were acquired through GlycoWorkbench software. The training, validation, and comprehensive metric evaluation of machine learning models are performed on Orange software. In model evaluation, logistic regression (LR), support vector machine (SVM), random forest (RF), and gradient boosting (GB) were employed. A total of 150 samples were randomly divided into a training set and a validation set at a ratio of 7:3, with each set containing an equal proportion of samples from the three cohorts. Furthermore, stratified 10‐fold cross‐validation was adopted to enhance the model's generalization ability and data utilization, and AUC, classification accuracy, F1 score, precision, specificity, and recall were used as comprehensive evaluation metrics for the model. The GB model was selected as the final diagnostic model. Based on one‐to‐rest and one‐to‐one strategies, the GB model was employed to preliminarily conduct classification comparisons at different levels for the three cohort samples, and evaluation was performed using AUC, accuracy, recall, and specificity. Finally, the classification performance of the key N‐glycans selected was also evaluated using AUC, accuracy, recall, and specificity.

The parameter details for the optimized Gradient Boosting model are as follows:

- Method: Extreme Gradient Boosting (xgboost)
- Number of trees: 500; Learning rate: 0.1
- Lambda: 1
- Limit depth of individual trees: 3
- Fraction of training instances: 0.7; Fraction of features for each tree: 0.7

The particle size and pore diameter of materials were measured using Nano Measurer software, with results presented as mean ± standard deviation (SD). Most data images, including material characterization plots, mass spectrometry images, radar charts, ROC curves, and probability distribution plots, were generated using Origin software (Origin 2018). Principal component analysis and a heat map were conducted with MetaboAnalyst 5.0. (https://www.metaboanalyst.ca/). The CV plots, bar charts, line charts, and box plots were analyzed and plotted using GraphPad Prism 8 software. The Pearson correlation coefficients were calculated using GraphPad Prism's analysis module with a two‐tailed test and a 95% confidence interval. One‐way ANOVA was used to calculate p‐values in bar charts of between‐group and within‐group repeatability. The Welch's *t*‐test was used to calculate the p‐value in the violin plots, which characterizes the significant differences between groups based on different N‐glycans. ns, *p* > 0.05; ^*^, *p* < 0.05; ^**^, *p* < 0.01; ^***^, *p* < 0.001; ^****^, *p* < 0.0001.

Accuracy, recall, and specificity are calculated based on actual states versus predicted states, encompassing true negatives (TN), false negatives (FN), true positives (TP), and false positives (FP). Accuracy represents the proportion of correct predictions among all predicted results. Recall represents the proportion of true positive cases correctly predicted as positive by the model. Specificity represents the proportion of true negative cases correctly predicted as negative by the model. The specific calculation formulas are as follows:

Accuracy=(TP+TN)/(TP+TN+FP+FN)

Recall=TP/(TP+FN)

Specificity=TN/(TN+FP)

## Ethics Approval

This study strictly followed the principles of the Helsinki Declaration and was approved by the Ethics Committee of the Institute of Biomedical Sciences of Fudan University (2019‐008).

## Conflicts of Interest

The authors declare no conflict of interest.

## Supporting information




**Supporting File**: advs74557‐sup‐0001‐SuppMat.docx.

## Data Availability

The data that support the findings of this study are available in the supplementary material of this article.
