# Supplementary material for: Hierarchical Channeled Graphitized Nanoarchitecture as a Diagnostic Platform for Maternal Fever Warning
Source: Adv Sci (Weinh). 2026 Feb 23;13(26):e21251. doi: 10.1002/advs.202521251 (PMC13159115; doi:10.1002/advs.202521251)
Supplement: Supplementary file 1 — Supporting File: advs74557‐sup‐0001‐SuppMat.docx. [file ADVS-13-e21251-s001.docx]

Supporting Information

**Hierarchical channeled graphitized nanoarchitecture as a diagnostic platform for maternal fever warning**

Yiwen Lin^‡^, Ning Li^‡^, Heyuhan Zhang, Xufang Hu^*^, Zhiqiang Liu^*^, Chunhui Deng^*^

**EXPERIMENTAL SECTION**

1. **Chemicals**

2,5-dihydroxybenzoic acid (DHB), sinapic acid (SA), chicken egg ovalbumin (OVA) were purchased from Sigma-Aldrich (USA). Polyvinyl pyrrolidone (PVP, average M.W. 8000, K15-19) was purchased from J&K Scientific. Styrene, sodium hydroxide (NaOH), potassium persulfate (K_2_S_2_O_8_), cobaltous nitrate hexahydrate (Co(NO_3_)_2_·6H_2_O), ammonium bicarbonate (ABC), trifluoroacetic acid (TFA), ammonia, methanol, N,N-Dimethylformamide (DMF), acetonitrile (ACN) and toluene were purchased from Sinopharm. Bovine plasma albumin (BSA), peptide N-glycosidase F (PNGase F, 15000U, 30 μL), 2-methylimidazole, tris(2-carboxyethyl)phosphine (TCEP), 2-chloroacetamide (CAA) were purchased from Adamas. Ethanol was purchased from General-Reagent. Dichloromethane (DCM) was purchased from Shanghai Dahe Chemicals Co., Ltd. The deionized water used in this work is provided after purification by the Milli-Q system (Millipore, Bedford, MA).

**2. Synthesis of ZIF-67 and graphitized carbon ZIF-67 (****GC-Z67)**

First, 50 mL of a methanol solution containing Co(NO_3_)_2_·6H_2_O (11.8 mg/mL) and PVP (10 mg/mL) was prepared. Then, 50 ml of a methanol solution containing 2-methylimidazole (13.3 mg/mL) was slowly poured into the above solution. After vigorous stirring for 1 min, the mixed solution was allowed to stand at room temperature for 12 h. The purple product was washed by ethanol for 3 times and dried under vacuum at 50 ℃ to acquire ZIF-67.

The ZIF-67 powder was put into a crucible and carbonized at 900 ℃ for 1.5 h in a nitrogen atmosphere to prepare GC-Z67.

**3. Characterizations**

Field emission scanning electron microscopy (SEM) was performed on Thermo scientific Apreo 2S (USA). Field emission transmission electron microscope (FETEM) images of materials were acquired on FEI Tecnai G2 F20 S-Twin at 200 kV (USA). Elemental mapping analysis of HPGC-Z67 was taken on a Tecnai G2 F20 S-Twin microscope (America) at 200 kV. Raman spectrum test was performed on a Hobria Jobin Yvon XploRA micro-Raman system with 532 nm laser and 4.6 cm^-1^ resolution. Powder X-ray diffraction (XRD) patterns were obtained on Bruker D2 PHASER X-ray polycrystalline diffractometer by Cu Kα rediation (40 kV, 40 mA). Nitrogen adsorption/desorption isotherm test was conducted on a Autosorb-IQ analyzer (USA).

**4. Sample Collection and Storage**

All 150 plasma samples were collected from Obstetrics & Gynecology Hospital of Fudan University. The plasma samples were divided into three groups: HCs (50 individuals) included women who received epidural analgesia but were not febrile (<37.5°C); ERMFs (50 individuals) included women with epidural analgesia-induced fever (>37.5°C) at delivery; CAMs (50 individuals) included women with fever at delivery (>37.5°C) due to chorioamnionitis.

This work strictly adhered to the principles of the Declaration of Helsinki, complied with the Code of Ethics of the World Medical Association, and was approved by the Ethics Committee (No. 2022-160). In addition, the plasma samples involved in this study were consented and authorised by all patients and volunteers. All plasma samples were stored in a -80 ℃ refrigerator prior to pre-processing.

**5. Preparation of OVA Digest**

OVA standard protein solution was prepared at a concentration of 1 μg/μL using 25 mM ABC buffer solution as solvent. The solution was incubated at 100 ℃ for 10 min to induce protein denaturation and subsequently cooled to room temperature. 1 μg of PNGase F enzyme was added to 200 μL of protein solution. The above mixed solution was incubated overnight at 37 ℃ and 800 rpm and N-glycans were released. The OVA digests obtained were preserved at -20 ℃ for subsequent use.

**6. MALDI-TOF MS Analysis**

1 μL DHB solution (TFA/ACN/H_2_O=0.1/50/49.9, 15 mg mL^-1^) and 2 μL N-glycan eluate were successively dripped onto a MALDI plate and air dried. The organic matrix used for protein analysis was SA solution (TFA/ACN/H_2_O=0.1/50/49.9, 15 mg mL^-1^), and other operations were performed as above. The UlrafleXtreme MALDI-TOF/TOF MS (Bruker Daltonic, Germany) was equipped with a 335 nm Nd:YAG laser with a laser intensity and frequency of 90% and 2000 Hz, respectively. The MALDI-TOF MS spectra of N-glycans were acquired in reflector positive mode, and protein spectra were obtained in linear positive mode. Quality control samples consist of six mixed plasma digests containing HC, ERMF, and CAM. A QC sample is interleaved with the preprocessing and enrichment procedures for every 10 serum samples, tested concurrently with the actual samples under the same batch and identical experimental conditions. Prior to MALDI-TOF MS detection, QC points are evenly distributed on the plate after every 10 plasma N-glycan sample points, totaling 15 points.

**SUPPLEMENTARY FIGURES**

**Figure S1.** SEM images of (a, b) ZIF-67 and (c) GC-Z67.

**Figure S2.** TEM images of (a) ZIF-67, (b) GC-Z67, (c)HP-Z67 and (d) HPGC-Z67.

**Figure S3.** Raman spectra of (a) HP-Z67 and (b) HPGC-Z67.

**Figure S4.** Nitrogen adsorption/desorption isotherms of (a) HP-Z67 and (c) ZIF-67; and pore size distribution curve of (b) HP-Z67 and (d) ZIF-67.

**Figure S5.** Signal peak intensities of 25 N-glycans enriched from OVA digests in three between-group reproducibility tests (n=3).

**Figure S6.** Signal peak intensities of 25 N-glycans enriched from OVA digests in three within-group reproducibility tests (n=3).

**Figure S7.** MALDI-TOF MS analysis of N-glycans enriched from OVA digestion using ZIF-67, GC-Z67, HP-Z67, and HPGC-Z67. The N-glycan signal peaks are labelled by quadrangular stars, and details of the N-glycans are displayed in Table S1.

**Figure S8.** MALDI-TOF MS spectra of N-glycans enriched from 100 ng/μL, 10 ng/μL, 1 ng/μL, 0.5 ng/μL, 0.2 ng/μL, and 0.1 ng/μL OVA digests using GC-Z67. The N-glycan signal peaks are labelled by quadrangular stars, and details of the N-glycans are displayed in Table S1.

**Figure S9.** MALDI-TOF MS analysis of N-glycans from standard mixtures of OVA digest, OVA, and BSA with a mass ratio of 1: 500: 500. MS spectra of N-glycans (a) and proteins (b) were acquired without enrichment process. MS spectra of N-glycans (c) and proteins (d) were obtained after HPGC-Z67 enrichment. The N-glycan signal peaks are labelled by quadrangular stars, and more detailed information of N-glycans is shown in Table S1.

**Figure S10.** (a) Signal peak intensities of 46 N-glycans enriched from plasma digests in three between-group reproducibility tests (n=3). (b) Signal peak intensities of 47 N-glycans enriched from plasma digests in three within-group reproducibility tests (n=3).

**Figure S11.** (a) The number of N-glycans enriched by GC-Z67 and their intensity ratio relative to the total signal across three parallel experiments at different volumes of plasma undergo digest. (b) The number of N-glycans enriched by HPGC-Z67 and their intensity ratio relative to the total signal across three parallel experiments at different volumes of plasma undergo digest. A fixed volume of 20 μL of the 10 μg/μL material suspension was used to investigate the effect of N-glycan to carbon material ratio on enrichment efficiency.

**Figure S12.** The number of N-glycans enriched by HPGC-Z67 and their intensity ratio relative to the total signal across three parallel experiments at different (a) enrichment times and (b) elution times.

**Figure S13.** The (a) number and (b) intensity ratio of N-glycans signals from three parallelly extracted N-glycan profiles in 15 QC points.

**Figure S14.** The PCA plot of 15 QC samples and all plasma samples (PS).

**Figure S15.** The (a) heatmap and (b) PCA plot of 59 plasma N-glycan signals from HC/CAM/ERMF.

**Figure S16.** ROC curves for four machine learning models classifying three cohorts based on 59 N-glycans in the (a) training and (b) validation sets. (c) Radar chart for the comprehensive metric evaluation of the validation set.

**Figure S17.** The GB model's ROC curves for classifying three cohorts based on 59 N-glycans across (a) training and (b) validation sets in one-vs-rest strategy. Corresponding confusions for (c) the training and (d) validation sets.

**Figure S18.** The sample-level plot depicting the probability predicted by 59 N-glycans-based GB model in the training sets for differentiating (a) HC/CAM, (b) HC/ERMG and (c) CAM/ERMF groups. The sample-level plot depicting the probability predicted by 59 N-glycans-based GB model in the validation sets for differentiating (d) HC/CAM, (e) HC/ERMG and (f) CAM/ERMF groups.

**Figure S19.** The GB model's ROC curves for classifying three cohorts based on 59 N-glycans across (a) training and (b) validation sets in one-vs-one strategy.

**Figure S20.** N-glycan screening based on model evaluation metrics and feature importance scores in the validation sets of (a) HC/Fever and (b) CAM/ERMF.

**Figure S21.** (a) The sample-level plot depicting the probability predicted by Feature Set 1-based GB model in the validation sets for differentiating HC/Fever. (b) The sample-level plot depicting the probability predicted by Feature Set 2-based GB model in the validation sets for differentiating CAM/ERMF.

**Figure S22.** Violin plot analysis of Feature Set 1 in HC/Fever. ns, *p*>0.05; *, *p*<0.05; **, *p*<0.01; ***, *p*<0.001; ****, *p*<0.0001.

**Figure S23.** Violin plot analysis of Feature Set 2 in CAM/ERMF. ns, *p*>0.05; *, *p*<0.05; **, *p*<0.01; ***, *p*<0.001; ****, *p*<0.0001.

**Figure S24.** (a) Hierarchical Clustering of HC/CAM/ERMF Based on Feature Set 3. (b) The confusion matrix for the GB model predicting HC/CAM/ERMF based on Feature Set 3 in the validation set.

**SUPPLEMENTARY TABLES**

**Table S1.** The detailed information of the identified N-glycans from OVA digest by HPGC-Z67. Potential N-glycan structures were searched from GlycomeDB using the GlycoWorkbench software through the m/z of oligosaccharides in positive ion mode. The capital “H” and “N” in “Composition” column represent hexose and N-acetylglucosamine, respectively.

| No. | m/z | Charge Carrier | Composition |
| --- | --- | --- | --- |
| 1 | 933.73 | [M+Na]^+^ | H3N2 |
| 2 | 1095.45 | [M+Na]^+^ | H4N2 |
| 3 | 1136.33 | [M+Na]^+^ | H3N3 |
| 4 | 1257.54 | [M+Na]^+^ | H5N2 |
| 5 | 1298.64 | [M+Na]^+^ | H4N3 |
| 6 | 1339.69 | [M+Na]^+^ | H3N4 |
| 7 | 1419.76 | [M+Na]^+^ | H6N2 |
| 8 | 1460.82 | [M+Na]^+^ | H5N3 |
| 9 | 1501.90 | [M+Na]^+^ | H4N4 |
| 10 | 1542.96 | [M+Na]^+^ | H3N5 |
| 11 | 1560.72 | [M+H]^+^ | H7N2 |
| 12 | 1582.96 | [M+Na]^+^ | H7N2 |
| 13 | 1622.45 | [M+Na]^+^ | H6N3 |
| 14 | 1663.08 | [M+Na]^+^ | H5N4 |
| 15 | 1704.14 | [M+Na]^+^ | H4N5 |
| 16 | 1745.21 | [M+Na]^+^ | H3N6 |
| 17 | 1866.34 | [M+Na]^+^ | H5N5 |
| 18 | 1907.40 | [M+Na]^+^ | H4N6 |
| 19 | 1948.47 | [M+Na]^+^ | H3N7 |
| 20 | 2028.55 | [M+Na]^+^ | H6N5 |
| 21 | 2069.55 | [M+Na]^+^ | H5N6 |
| 22 | 2110.69 | [M+Na]^+^ | H4N7 |
| 23 | 2151.77 | [M+Na]^+^ | H3N8 |
| 24 | 2313.02 | [M+Na]^+^ | H4N8 |
| 25 | 2476.24 | [M+Na]^+^ | H5N8 |

**Table S2**. Performance comparison of HPGC-Z67 with other reported materials.

| Materials used | detection limit | Selectivity | Sample | detected glycans | Ref. |
| --- | --- | --- | --- | --- | --- |
| Bacterial cellulose | 667 fmol/μL | 1:100 | 10 μL serum | 79 | [S1] |
| carbon matrix | 1 ng/μL | 1 : 300 : 300 | serum containing 500 μg protein | 23 | [S2] |
| HPC-Ce/Fe | 1 ng/μL | 1:400:400 | 15 μL serum | 36 | [S3] |
| MMC-Fe | 1 ng/µL | 1 : 300 : 300 | serum containing 500 μg protein | 22. | [S4] |
| C-graphene@mSiO_2_ | － | 100:1 | 400 nL serum | 48 | [S5] |
| IPC-O | 2 ng/μL | 1:500:500 | 5 μL serum | 49 | [S6] |
| HPGC-Z67 | 0.1 ng/μL | 1:500:500 | 15 μL plasma | 59 | This work |

**Table S3.** Detailed information on 71 plasma N-glycans was obtained from three cohorts (HC/CAM/ERMF) by HPGC-Z67. Potential N-glycan structures were searched from GlycomeDB using the GlycoWorkbench software through the m/z of oligosaccharides in positive ion mode. The capital “H”, “N”, “F” and “S” in “Composition” column represent mannose/galactose, N-acetylglucosamine, fucose and sialic acid, respectively.

| No. | m/z | Charge Carrier | Composition | Gorup |
| --- | --- | --- | --- | --- |
| 1 | 933.32 | [M+Na]^+^ | H3N2 | HC/CAM |
| 2 | 1079.38 | [M+Na]^+^ | H3N2F1 | HC/ERMF/CAM |
| 3 | 1095.37 | [M+Na]^+^ | H4N2 | HC/ERMF/CAM |
| 4 | 1136.40 | [M+Na]^+^ | H3N3 | HC/ERMF/CAM |
| 5 | 1257.42 | [M+Na]^+^ | H5N2 | HC/ERMF/CAM |
| 6 | 1282.45 | [M+Na]^+^ | H3N3F1 | HC/ERMF/CAM |
| 7 | 1298.45 | [M+Na]^+^ | H4N3 | HC/ERMF/CAM |
| 8 | 1339.48 | [M+Na]^+^ | H3N4 | HC/ERMF/CAM |
| 9 | 1403.48 | [M+Na]^+^ | H5N2F1 | HC/CAM |
| 10 | 1419.48 | [M+Na]^+^ | H6N2 | HC/ERMF/CAM |
| 11 | 1444.51 | [M+Na]^+^ | H4N3F1 | HC/ERMF/CAM |
| 12 | 1460.50 | [M+Na]^+^ | H5N3 | HC/ERMF/CAM |
| 13 | 1485.53 | [M+Na]^+^ | H3N4F1 | HC/ERMF/CAM |
| 14 | 1501.53 | [M+Na]^+^ | H4N4 | HC/ERMF/CAM |
| 15 | 1542.56 | [M+Na]^+^ | H3N5 | HC/ERMF/CAM |
| 16 | 1581.53 | [M+Na]^+^ | H7N2 | HC/ERMF/CAM |
| 17 | 1606.56 | [M+Na]^+^ | H5N3F1 | HC/ERMF/CAM |
| 18 | 1608.59 | [M+H]^+^ | H3N4S1 | HC/ERMF/CAM |
| 19 | 1622.56 | [M+Na]^+^ | H6N3 | HC/ERMF/CAM |
| 20 | 1631.59 | [M+Na]^+^ | H3N4F2 | ERMF/CAM |
| 21 | 1647.59 | [M+Na]^+^ | H4N4F1 | HC/ERMF/CAM |
| 22 | 1663.58 | [M+Na]^+^ | H5N4 | HC/ERMF/CAM |
| 23 | 1688.61 | [M+Na]^+^ | H3N5F1 | HC/ERMF/CAM |
| 24 | 1704.61 | [M+Na]^+^ | H4N5 | HC/ERMF/CAM |
| 25 | 1743.58 | [M+Na]^+^ | H8N2 | HC/ERMF/CAM |
| 26 | 1745.63 | [M+Na]^+^ | H3N6 | HC/ERMF/CAM |
| 27 | 1751.60 | [M+Na]^+^ | H5N3S1 | HC/ERMF/CAM |
| 28 | 1768.61 | [M+Na]^+^ | H6N3F1 | HC/ERMF/CAM |
| 29 | 1793.65 | [M+Na]^+^ | H4N4F2 | HC/ERMF/CAM |
| 30 | 1809.64 | [M+Na]^+^ | H5N4F1 | HC/ERMF/CAM |
| 31 | 1825.63 | [M+Na]^+^ | H6N4 | HC/ERMF/CAM |
| 32 | 1850.67 | [M+Na]^+^ | H4N5F1 | HC/ERMF/CAM |
| 33 | 1866.66 | [M+Na]^+^ | H5N5 | HC/ERMF/CAM |
| 34 | 1891.69 | [M+Na]^+^ | H3N6F1 | HC/ERMF/CAM |
| 35 | 1905.63 | [M+Na]^+^ | H9N2 | HC/ERMF/CAM |
| 36 | 1907.69 | [M+Na]^+^ | H4N6 | HC/ERMF/CAM |
| 37 | 1926.73 | [M+H]^+^ | H3N7 | HC/ERMF/CAM |
| 38 | 1954.68 | [M+Na]^+^ | H5N4S1 | HC/ERMF/CAM |
| 39 | 1971.69 | [M+Na]^+^ | H5N4F2 | HC/ERMF/CAM |
| 40 | 1974.74 | [M+Na]^+^ | H6N4F1 | HC/ERMF/CAM |
| 41 | 1995.70 | [M+Na]^+^ | H4N5S1 | HC/ERMF/CAM |
| 42 | 2012.72 | [M+Na]^+^ | H5N5F1 | HC/ERMF/CAM |
| 43 | 2018.64 | [M+Na]^+^ | H4N4F1S1 | HC/ERMF/CAM |
| 44 | 2028.71 | [M+Na]^+^ | H6N5 | HC/ERMF/CAM |
| 45 | 2037.75 | [M+Na]^+^ | H3N6F2 | ERMF |
| 46 | 2053.75 | [M+Na]^+^ | H4N6F1 | HC/ERMF/CAM |
| 47 | 2067.69 | [M+Na]^+^ | H10N2 | HC |
| 48 | 2069.74 | [M+Na]^+^ | H5N6 | HC/ERMF/CAM |
| 49 | 2101.76 | [M+Na]^+^ | H5N4F3 | HC/ERMF/CAM |
| 50 | 2110.77 | [M+Na]^+^ | H4N7 | HC |
| 51 | 2151.79 | [M+Na]^+^ | H3N8 | HC/ERMF/CAM |
| 52 | 2158.78 | [M+Na]^+^ | H5N5F2 | HC/ERMF/CAM |
| 53 | 2174.77 | [M+Na]^+^ | H6N5F1 | HC/ERMF/CAM |
| 54 | 2215.80 | [M+Na]^+^ | H5N6F1 | CAM |
| 55 | 2221.72 | [M+Na]^+^ | H4N5F1S1 | HC/ERMF/CAM |
| 56 | 2231.79 | [M+Na]^+^ | H6N6 | HC/ERMF/CAM |
| 57 | 2240.83 | [M+Na]^+^ | H3N7F2 | HC/ERMF/CAM |
| 58 | 2245.77 | [M+Na]^+^ | H5N4S2 | HC/ERMF/CAM |
| 59 | 2288.84 | [M+Na]^+^ | H4N5F2S1 | HC/ERMF/CAM |
| 60 | 2305.24 | [M+Na]^+^ | H5N5F3 | HC/ERMF/CAM |
| 61 | 2320.83 | [M+Na]^+^ | H6N5F2 | HC |
| 62 | 2328.85 | [M+Na]^+^ | H3N6F2S1 | HC/ERMF/CAM |
| 63 | 2332.84 | [M+Na]^+^ | H3N9 | HC/ERMF/CAM |
| 64 | 2361.86 | [M+Na]^+^ | H5N6F2 | HC/ERMF/CAM |
| 65 | 2386.89 | [M+Na]^+^ | H3N7F3 | HC/ERMF |
| 66 | 2393.85 | [M+Na]^+^ | H7N6 | HC/ERMF |
| 67 | 2427.89 | [M+H]^+^ | H5N5F2S1 | HC/ERMF/CAM |
| 68 | 2433.89 | [M+Na]^+^ | H6N4S2 | HC/ERMF/CAM |
| 69 | 2437.92 | [M+H]^+^ | H4N8F1 | HC/ERMF/CAM |
| 70 | 2466.89 | [M+Na]^+^ | H6N5F3 | HC/ERMF |
| 71 | 2473.88 | [M+Na]^+^ | H3N6F1S2 | HC |

**Table S4.** The detailed information of all plasma samples in this work. HC denotes healthy controls; ERMF denotes maternal fever associated with epidural analgesia; CAM denotes maternal fever induced by chorioamnionitis. BMI represents body mass index, calculated as weight (kg) divided by height (m) squared.

| No. | Sample | Age | BMI | Highest Temperature |
| --- | --- | --- | --- | --- |
| 1 | HC | 30 | 27.56 | 37.1 |
| 2 | HC | 34 | 30.31 | 36.5 |
| 3 | HC | 33 | 28.13 | 37.0 |
| 4 | HC | 31 | 28.71 | 37.3 |
| 5 | HC | 33 | 36.95 | 37.0 |
| 6 | HC | 37 | 25.95 | 37.2 |
| 7 | HC | 30 | 25.39 | 37.2 |
| 8 | HC | 31 | 26.12 | 37.0 |
| 9 | HC | 33 | 32.32 | 36.8 |
| 10 | HC | 32 | 26.23 | 37.2 |
| 11 | HC | 37 | 23.53 | 37.0 |
| 12 | HC | 27 | 27.18 | 37.0 |
| 13 | HC | 35 | 21.29 | 37.2 |
| 14 | HC | 32 | 22.49 | 37.1 |
| 15 | HC | 30 | 31.88 | 37.2 |
| 16 | HC | 36 | 32.08 | 37.3 |
| 17 | HC | 30 | 25.71 | 37.2 |
| 18 | HC | 35 | 31.64 | 37.3 |
| 19 | HC | 37 | 27.19 | 37.1 |
| 20 | HC | 28 | 25.20 | 37.3 |
| 21 | HC | 23 | 25.00 | 37.4 |
| 22 | HC | 32 | 20.96 | 37.4 |
| 23 | HC | 27 | 29.31 | 37.2 |
| 24 | HC | 35 | 23.65 | 37.1 |
| 25 | HC | 28 | 24.35 | 37.4 |
| 26 | HC | 28 | 23.38 | 37.3 |
| 27 | HC | 30 | 30.62 | 37.4 |
| 28 | HC | 32 | 28.47 | 37.2 |
| 29 | HC | 28 | 22.03 | 37.0 |
| 30 | HC | 31 | 25.71 | 37.4 |
| 31 | HC | 32 | 26.83 | 37.4 |
| 32 | HC | 29 | 23.60 | 37.4 |
| 33 | HC | 26 | 25.33 | 37.1 |
| 34 | HC | 28 | 29.04 | 37.2 |
| 35 | HC | 26 | 29.06 | 36.5 |
| 36 | HC | 32 | 23.71 | 37.2 |
| 37 | HC | 32 | 34.89 | 37.2 |
| 38 | HC | 35 | 23.18 | 37.3 |
| 39 | HC | 30 | 23.03 | 37.3 |
| 40 | HC | 34 | 24.22 | 37.1 |
| 41 | HC | 32 | 24.60 | 37.3 |
| 42 | HC | 36 | 23.22 | 37.1 |
| 43 | HC | 27 | 25.23 | 37.4 |
| 44 | HC | 34 | 31.51 | 37.4 |
| 45 | HC | 35 | 20.76 | 36.8 |
| 46 | HC | 31 | 26.36 | 37.0 |
| 47 | HC | 33 | 27.70 | 37.1 |
| 48 | HC | 35 | 29.28 | 36.9 |
| 49 | HC | 34 | 27.73 | 37.0 |
| 50 | HC | 34 | 26.84 | 37.2 |
| 51 | ERMF | 33 | 24.40 | 37.6 |
| 52 | ERMF | 28 | 26.04 | 37.8 |
| 53 | ERMF | 32 | 27.19 | 38.0 |
| 54 | ERMF | 36 | 20.70 | 37.8 |
| 55 | ERMF | 28 | 22.49 | 38.1 |
| 56 | ERMF | 33 | 26.77 | 38.1 |
| 57 | ERMF | 43 | 27.51 | 37.7 |
| 58 | ERMF | 30 | 28.04 | 37.5 |
| 59 | ERMF | 31 | 26.27 | 38.2 |
| 60 | ERMF | 38 | 30.48 | 37.8 |
| 61 | ERMF | 29 | 25.33 | 37.7 |
| 62 | ERMF | 36 | 26.55 | 38.0 |
| 63 | ERMF | 37 | 27.54 | 37.7 |
| 64 | ERMF | 33 | 23.71 | 38.0 |
| 65 | ERMF | 32 | 29.09 | 39.2 |
| 66 | ERMF | 30 | 26.24 | 38.0 |
| 67 | ERMF | 34 | 24.46 | 38.0 |
| 68 | ERMF | 32 | 27.35 | 38.0 |
| 69 | ERMF | 32 | 29.60 | 38.3 |
| 70 | ERMF | 33 | 30.00 | 38.4 |
| 71 | ERMF | 28 | 30.89 | 38.0 |
| 72 | ERMF | 34 | 22.37 | 38.0 |
| 73 | ERMF | 28 | 23.63 | 37.8 |
| 74 | ERMF | 32 | 27.34 | 38.0 |
| 75 | ERMF | 32 | 25.68 | 38.0 |
| 76 | ERMF | 29 | 30.85 | 38.2 |
| 77 | ERMF | 23 | 21.77 | 38.3 |
| 78 | ERMF | 39 | 25.10 | 38.0 |
| 79 | ERMF | 33 | 24.03 | 38.1 |
| 80 | ERMF | 36 | 25.71 | 37.9 |
| 81 | ERMF | 27 | 22.58 | 38.3 |
| 82 | ERMF | 34 | 24.03 | 37.8 |
| 83 | ERMF | 31 | 28.09 | 38.1 |
| 84 | ERMF | 31 | 23.43 | 38.0 |
| 85 | ERMF | 29 | 23.58 | 37.8 |
| 86 | ERMF | 27 | 27.65 | 38.0 |
| 87 | ERMF | 31 | 31.44 | 37.7 |
| 88 | ERMF | 30 | 27.47 | 38.5 |
| 89 | ERMF | 35 | 25.40 | 38.0 |
| 90 | ERMF | 28 | 30.10 | 38.1 |
| 91 | ERMF | 27 | 29.34 | 37.8 |
| 92 | ERMF | 34 | 28.48 | 38.1 |
| 93 | ERMF | 29 | 24.60 | 38.2 |
| 94 | ERMF | 27 | 27.57 | 38.0 |
| 95 | ERMF | 33 | 29.80 | 37.7 |
| 96 | ERMF | 29 | 29.10 | 37.6 |
| 97 | ERMF | 31 | 28.81 | 38.1 |
| 98 | ERMF | 27 | 41.50 | 37.8 |
| 99 | ERMF | 28 | 25.46 | 38.0 |
| 100 | ERMF | 32 | 26.05 | 37.9 |
| 101 | CAM | 30 | 28.07 | 38.1 |
| 102 | CAM | 31 | 27.00 | 38.7 |
| 103 | CAM | 31 | 33.91 | 37.6 |
| 104 | CAM | 31 | 26.75 | 38.3 |
| 105 | CAM | 26 | 28.93 | 37.7 |
| 106 | CAM | 34 | 26.50 | 38.0 |
| 107 | CAM | 30 | 29.68 | 37.8 |
| 108 | CAM | 35 | 32.04 | 38.2 |
| 109 | CAM | 21 | 22.55 | 38.2 |
| 110 | CAM | 33 | 34.37 | 38.0 |
| 111 | CAM | 35 | 28.93 | 38.0 |
| 112 | CAM | 33 | 27.60 | 38.3 |
| 113 | CAM | 32 | 24.09 | 38.0 |
| 114 | CAM | 34 | 25.09 | 38.2 |
| 115 | CAM | 29 | 24.31 | 37.9 |
| 116 | CAM | 34 | 25.71 | 37.9 |
| 117 | CAM | 37 | 28.32 | 38.5 |
| 118 | CAM | 26 | 25.06 | 37.8 |
| 119 | CAM | 29 | 22.46 | 38.0 |
| 120 | CAM | 31 | 27.85 | 38.3 |
| 121 | CAM | 33 | 29.85 | 38.1 |
| 122 | CAM | 30 | 26.20 | 38.6 |
| 123 | CAM | 31 | 24.72 | 38.3 |
| 124 | CAM | 33 | 29.05 | 37.9 |
| 125 | CAM | 29 | 27.23 | 38.1 |
| 126 | CAM | 33 | 26.00 | 37.8 |
| 127 | CAM | 30 | 29.58 | 38.1 |
| 128 | CAM | 28 | 23.21 | 38.0 |
| 129 | CAM | 28 | 30.84 | 38.4 |
| 130 | CAM | 31 | 28.83 | 38.0 |
| 131 | CAM | 28 | 25.03 | 38.0 |
| 132 | CAM | 30 | 21.63 | 38.2 |
| 133 | CAM | 31 | 30.48 | 38.1 |
| 134 | CAM | 32 | 26.85 | 37.9 |
| 135 | CAM | 28 | 26.64 | 38.1 |
| 136 | CAM | 28 | 26.57 | 38.0 |
| 137 | CAM | 30 | 25.14 | 38.0 |
| 138 | CAM | 35 | 29.98 | 38.1 |
| 139 | CAM | 31 | 28.37 | 38.2 |
| 140 | CAM | 32 | 29.46 | 38.6 |
| 141 | CAM | 31 | 27.47 | 38.1 |
| 142 | CAM | 30 | 24.46 | 37.8 |
| 143 | CAM | 30 | 31.40 | 37.8 |
| 144 | CAM | 35 | 31.93 | 38.0 |
| 145 | CAM | 30 | 26.08 | 38.0 |
| 146 | CAM | 30 | 28.28 | 39.0 |
| 147 | CAM | 28 | 28.14 | 38.1 |
| 148 | CAM | 34 | 27.34 | 38.0 |
| 149 | CAM | 29 | 27.34 | 38.6 |
| 150 | CAM | 30 | 26.77 | 38.3 |

**Table S5.** Comprehensive metrics for four machine learning models classifying three cohorts based on 59 N-glycans in the training and validation sets.

| **Training Set** | | | | | | |
| --- | --- | --- | --- | --- | --- | --- |
| Model | AUC | Accuracy | F1 score | Precision | Recall | Specificity |
| Logistic Regression | 0.802 | 0.686 | 0.683 | 0.685 | 0.686 | 0.843 |
| Random Forest | 0.941 | 0.819 | 0.818 | 0.820 | 0.819 | 0.924 |
| SVM | 0.976 | 0.914 | 0.913 | 0.916 | 0.914 | 0.957 |
| Gradient Boosting | 0.984 | 0.933 | 0.933 | 0.934 | 0.933 | 0.967 |

| **Validation Set** | | | | | | |
| --- | --- | --- | --- | --- | --- | --- |
| Model | AUC | Accuracy | F1 score | Precision | Recall | Specificity |
| Logistic Regression | 0.878 | 0.778 | 0.779 | 0.788 | 0.778 | 0.889 |
| Random Forest | 0.956 | 0.800 | 0.795 | 0.798 | 0.800 | 0.922 |
| SVM | 0.967 | 0.889 | 0.889 | 0.890 | 0.889 | 0.944 |
| Gradient Boosting | 0.973 | 0.911 | 0.909 | 0.913 | 0.911 | 0.956 |

**Table S6.** Comprehensive metrics of the GB model classifying three cohorts based on 59 N-glycans across the training and validation sets in one-vs-rest strategy.

| **Training Set** | | | | |
| --- | --- | --- | --- | --- |
| Group | AUC | Accuracy | Recall | Specificity |
| HC | 0.988 | 0.943 | 0.914 | 0.957 |
| ERMF | 0.995 | 0.962 | 0.943 | 0.971 |
| CAM | 0.990 | 0.943 | 0.914 | 0.957 |

| **Validation Set** | | | | |
| --- | --- | --- | --- | --- |
| Group | AUC | Accuracy | Recall | Specificity |
| HC | 0.953 | 0.911 | 0.800 | 0.967 |
| ERMF | 0.984 | 0.956 | 1.000 | 0.933 |
| CAM | 0.993 | 0.956 | 0.933 | 0.967 |

**Table S7.** Comprehensive metrics of the GB model classifying three cohorts based on 59 N-glycans across the training and validation sets in one-vs-one strategy.

| **Training Set** | | | | |
| --- | --- | --- | --- | --- |
| Group | AUC | Accuracy | Recall | Specificity |
| CAM-ERMF | 0.993 | 0.943 | 0.943 | 0.943 |
| HC-CAM | 0.973 | 0.943 | 0.943 | 0.943 |
| HC-ERMF | 0.991 | 0.957 | 0.957 | 0.957 |

| **Validation Set** | | | | |
| --- | --- | --- | --- | --- |
| Group | AUC | Accuracy | Recall | Specificity |
| CAM-ERMF | 1.000 | 0.967 | 0.967 | 0.967 |
| HC-CAM | 0.973 | 0.967 | 0.967 | 0.967 |
| HC-ERMF | 1.000 | 0.967 | 0.967 | 0.967 |

**Table S8.** The detailed information of Feature Set 1 and Feature Set 2.

| **HC-Fever** | | | | | | | |
| --- | --- | --- | --- | --- | --- | --- | --- |
| **Feature Set 1** | *p*-value | Feature  Importance  Score | Charge Carrier | Composition | High  Branching | Fucosylation | Sialylation |
| 2305.24 | 4.60E-11 | 0.069 | [M+Na]^+^ | H5N5F3 | Yes | Yes | No |
| 1751.60 | 7.30E-03 | 0.058 | [M+Na]^+^ | H5N3S1 | No | No | Yes |
| 1866.66 | 5.02E-03 | 0.055 | [M+Na]^+^ | H5N5 | Yes | No | No |
| 1688.61 | 1.55E-02 | 0.042 | [M+Na]^+^ | H3N5F1 | Yes | Yes | No |
| 1793.65 | 5.21E-06 | 0.036 | [M+Na]^+^ | H4N4F2 | No | Yes | No |

| **CAM-ERMF** | | | | | | | |
| --- | --- | --- | --- | --- | --- | --- | --- |
| **Feature Set 2** | *p*-value | Feature  Importance  Score | Charge Carrier | Composition | High  Branching | Fucosylation | Sialylation |
| 2305.24 | 4.64E-19 | 0.128 | [M+Na]^+^ | H5N5F3 | Yes | Yes | No |
| 1809.64 | 4.25E-03 | 0.115 | [M+Na]^+^ | H5N4F1 | No | Yes | No |
| 1793.65 | 9.97E-21 | 0.064 | [M+Na]^+^ | H4N4F2 | No | Yes | No |
| 1282.45 | 1.41E-05 | 0.034 | [M+Na]^+^ | H3N3F1 | No | Yes | No |
| 1745.63 | 8.93E-03 | 0.033 | [M+Na]^+^ | H3N6 | Yes | No | No |

**Table S9.** Comprehensive metrics of the GB model classifying HC/Fever based on Feature Set 1 across the training and validation sets.

| **HC-Fever** | | | | |
| --- | --- | --- | --- | --- |
|  | AUC | Accuracy | Recall | Specificity |
| Training Set | 0.951 | 0.905 | 0.800 | 0.957 |
| Validation Set | 0.956 | 0.933 | 0.867 | 0.967 |

**Table S10.** Comprehensive metrics of the GB model classifying CAM/ERMF based on Feature Set 2 across the training and validation sets.

| **CAM-ERMF** | | | | |
| --- | --- | --- | --- | --- |
|  | AUC | Accuracy | Recall | Specificity |
| Training Set | 0.991 | 0.943 | 0.943 | 0.943 |
| Validation Set | 1.000 | 1.000 | 1.000 | 1.000 |

**Table S11.** Comprehensive metrics of the GB model classifying three cohorts based on Feature Set 3 in the training and validation sets.

| **HC-CAM-ERMF** | | | | |
| --- | --- | --- | --- | --- |
|  | AUC | Accuracy | Recall | Specificity |
| Training Set | 0.965 | 0.867 | 0.867 | 0.933 |
| Validation Set | 0.914 | 0.800 | 0.800 | 0.900 |

**References**

[S1] M. Wu, Q. Zhang, X. Zhou, S. Kong, H. Zhao, M. Liu, P. Yang, W. Cao, An ultrafast and highly efficient enrichment method for both N-glycopeptides and N-glycans by bacterial cellulose. *Anal. Chim. Acta*, **2020**, *1140*, 60–68.

[S2] Y. Wu, Y. Chen, H. Chen, C. Yang, X. Shen, C. Deng, N. Sun, H. Wu, Probing serum N-glycan patterns for rapid and precise detection of Crohn's disease. *Chem. Commun*. **2021**, *57*, 11362–11365.

[S3] Y. Lin, Y. Chen, Y. Wu, C. Deng, S. Jiang, N. Sun, Branched Hierarchical Porous Carbon Enables Ultrasensitive Detection of Serum Glycans for Comprehensive Assessment of Urological Cancers. *Small methods* **2025**, *9*, e2402033.

[S4] Y. Lin, Y. Chen, C. Deng, N. Sun, Integration of resol/block-copolymer carbonization and machine learning: A convenient approach for precise monitoring of glycan-associated disorders. *Chin. Chem. Lett.* **2024**, *35*, 109813.

[S5] N. Sun, C. Deng, Y. Li, X. Zhang, Highly selective enrichment of N-linked glycan by carbon-functionalized ordered graphene/mesoporous silica composites. *Anal. Chem.* **2014**, *86*, 2246–2250.

[S6] J.Wang, J. Liu, M. Li, Y. Wang, Q. Man, H. Zhang, L. H. Huang, X. Zhang, Novel Three-Dimensional Hierarchical Porous Carbon Probe for the Discovery of N-Glycan Biomarkers and Early Hepatocellular Carcinoma Detection. *Anal. Chem.* **2023**, *95*, 10231–10240.
